# Stakeholders’ Views on Mobile Applications to Deliver Infant and Toddler Feeding Education to Latina Mothers of Low Socioeconomic Status

**DOI:** 10.3390/nu13082569

**Published:** 2021-07-27

**Authors:** Alexandra L. MacMillan Uribe, Hannah G. Rudt, Tashara M. Leak

**Affiliations:** Division of Nutritional Sciences, Cornell University, 244 Garden Avenue, Ithaca, NY 14853, USA; hgr26@cornell.edu (H.G.R.); tml226@cornell.edu (T.M.L.)

**Keywords:** digital technologies, mHealth, mobile application, infant feeding, toddler feeding, disparities, Hispanic, Latinx, qualitative research, nutrition education

## Abstract

Infant- and toddler-feeding (ITF) practices are critical to long-term health and chronic disease prevention. Using mobile applications (apps) to promote desirable ITF practices shows promise for overcoming challenges of in-person education. However, the viability of ITF apps for Latina mothers of low-socioeconomic status (SES) remains unclear. The objective of this study was to characterize stakeholders’ views on Latina mothers’ capability, motivation, and barriers to using ITF apps. New York City-based health professionals who frequently engage with Latina mothers of low SES completed in-depth interviews. Directed content analysis was used to identify themes through theoretical and inductive codes. Participants included dietitians, nutrition educators, and physicians (n = 17). The following themes were identified: (1) Most Latina mothers of low-SES are tech-savvy (i.e., high capability and experience using smartphones and apps); (2) Apps are an appealing way to deliver ITF education; (3) There are challenges to using apps that must be carefully considered for ITF education development. Overall, ITF apps are a viable option as skills and use appear high among Latina mothers. Key considerations for app development include targeted app promotion; detailed instructions for obtaining and using app; more visuals, less text for low literacy and multiple dialects; making key features available offline.

## 1. Introduction

Racial and ethnic disparities in obesity begin early in life, disproportionately impacting the Latinx community (Latinx is a gender-neutral term describing a person of Latin American origin or descent). Latinx infants and toddlers (3–24 months old) from low-socioeconomic status (SES) households have a significantly higher rate (13.8%) of excess weight (defined as weight-for-length >97.7th percentile on sex-specific World Health Organization [WHO] growth standards) compared to their Black (11.9%) and White (11%) counterparts [1]. Excess weight during infancy and toddlerhood has adverse health consequences during childhood (e.g., pre-diabetes and high cholesterol) [2] and adulthood (e.g., increased risk of subsequent obesity, type 2 diabetes, and cardiovascular disease) [3,4].

Feeding practices (e.g., breast or formula feeding, complementary feeding, responsive feeding, and transition to family foods) influence the risk of excess weight gain during infancy and toddlerhood and, in turn, obesity later in life [4,5]. Exclusive breastfeeding is protective from excess weight gain during infancy and later obesity [6]. Despite high rates of breastfeeding initiation, Latina women in the United States (US) are less likely to exclusively breastfeed compared to Black or White mothers [7]. Diet quality, which influences weight gain, is of concern among Latinx infants and toddlers. One study using National Health and Nutrition Examination Survey data revealed that a significantly lower proportion of Latinx infants 6 to 11 months old consumed fruits and vegetables (excluding white potatoes) and a higher proportion consumed sweets (sweet bakery products, candy, or other desserts) compared to White infants [8]. Around 12 months of age, when children transition from infant foods (e.g., pureed or mashed foods) to foods consumed by the family, there is a drastic decline in diet quality among Latinx toddlers, characterized by decreased vegetable consumption and increased consumption of sweets and sugar-sweetened beverages [8].

Behavior-change interventions focused on infant and toddler feeding (ITF) practices have the potential to increase desirable feeding practices and prevent obesity [9]. To date, the majority of ITF interventions are designed for children over 2 years old and those targeting Latinx infants or toddlers are scarce [10]. The majority of ITF interventions for Latina mothers are in-person [11,12,13,14,15,16,17,18]. In-person interventions are susceptible to various challenges (e.g., scheduling, time constraints, and childcare for older children) [19] and can be resource- and time-intensive, making wider dissemination difficult [20].

Mobile health (mHealth) interventions present an opportunity to overcome the challenges of in-person education and are highly scalable, with a wide dissemination potential. mHealth is defined as, “medical and public health practice supported by mobile devices, such as mobile phones, patient monitoring devices, personal digital assistants, and other wireless devices,” by the Global Observatory for eHealth of the WHO [21]. mHealth is an attractive delivery method as it capitalizes on the ubiquity of smartphones and information-seeking behaviors of mothers: most US Latinx (regardless of SES) own smartphones (98%) [22] and mothers frequently use their smartphone to access parenting and child health information [23,24].

Mobile applications (apps) present a robust mHealth delivery platform that can make information more easily accessible, offer personalized information, and engage users in diverse ways (e.g., visuals, resource library, instructional videos, message board to connect with peers and professionals) [25]. Additionally, Latinx are more likely to use health apps compared to other race/ethnic groups and there is early qualitative evidence of acceptance of health apps among mothers participating in the Special Supplemental Nutrition Program for Women, Infants, and Children (WIC) [26,27]. To date, only two nutrition education apps for preschool aged children include Latina mothers of low SES, both of which are currently under development [28,29]. Neither of these interventions specifically target the Latina population (rather they target a broader WIC population) nor focus specifically on ITF education. There remains a critical need to understand the acceptability of using an app to deliver ITF education to Latina mothers. This information will be valuable in informing the development of a culturally tailored obesity prevention app intervention for infants and toddlers of Latina mothers of low SES.

The purpose of this study was to obtain insight on using an app to deliver ITF education to Latina mothers of low SES. Community stakeholders, namely health and nutrition professionals, were chosen as participants because they could provide important insight from working with Latina mothers of low SES with a variety of experiences. The primary research question that guided this work was, “What are community stakeholders’ views on using an app to deliver ITF education to Latina mothers of low SES?”.

## 2. Materials and Methods

### 2.1. Study Design

The research question was explored through semi-structured qualitative interviews. The interview guide was informed by the existing literature and the Capability, Opportunity, Motivation, and Behavior (COM-B) framework, part of the Behavior Change Wheel (Table 1) [30]. The COM-B framework represents a starting point for determining whether an intervention is feasible in a specified setting by identifying three critical components for behavior change: capability (individual’s physical and psychological ability to perform a behavior), opportunity (factors outside the individual’s control that prompt behavior), and motivation (brain processes that energize and direct behavior) [30].

To obtain face validity, the guide was reviewed by two researchers with expertise in community-based interventions, ascertaining that the questions and terminology were appropriate for the audience. Additionally, the interview guide was pilot tested with two graduate research assistants, both of whom have extensive experience with community- based research and one of whom is a registered dietitian. The pilot test resulted in small changes to wording and terminology. Open-ended questions and probing techniques were used, ensuring all relevant topics were explored.

### 2.2. Participants and Recruitment

Stakeholders who met the following criteria were eligible to participate: (1) ≥18 years old; (2) had ≥3 years of experience providing health or nutrition services to Latina mothers of low SES with infants or toddlers (6–24 months old); (3) provided health or nutrition services to Latina mothers of low SES regularly (≥1 time per week). Purposive maximum variation sampling was carried out targeting stakeholders with a variety of job titles (e.g., registered dietitians, physicians, and nutrition educators). Purposive maximum variation sampling is a strategic sampling technique to study a wide variety of participants to ensure a balanced perspective of a group’s collective views and experiences [31].

Stakeholders were recruited through community partner sites, clinics, and professional societies based in New York City, NY (NYC). The community partner sites included two Federally Qualified Healthcare Centers and two Cooperative Extension offices. Recruitment materials (email and flyer) included a link and a phone number to complete a consent form, screener survey, and a brief demographic and digital use survey either online (Qualtrics) or over the phone. Questions for the digital use survey were from the Pew Research Core Trends survey [32]. Eligible individuals also provided available times to be scheduled for an interview via phone or video conferencing (Zoom, version 5.6.4 [Zoom Video Communications, Inc., 2021, San Jose, CA, USA]). Participants received a $50 gift card incentive. The Cornell University Institutional Review Board approved this study, deeming it exempt from review.

### 2.3. Data Collection

Participants completed a screener, demographic, digital use survey prior to the interview (as described above). Video interviews were recorded using the video conferencing software recording features and phone interviews were audio recorded. All interviews were conducted by the primary researcher (A.L.M.U.) with one research assistant. The primary researcher took brief notes during the interview while one research assistant took notes of verbal inflections and non-verbal cues (for video interviews) and summarized points on each interview guide question (Table 1) and the main research question (stated above). At the time of the interviews, the interviewer (A.L.M.U.) was trained in qualitative methods through graduate-level coursework, mentorship from qualitative experts, and 12 years of qualitative research experience. The research assistants were trained by A.L.M.U., took an online qualitative methods course, and read relevant scientific articles.

### 2.4. Data Analysis

#### 2.4.1. Qualitative Analysis

Saturation of the data was defined as consistent responses or exhaustion of possible responses to the main research question [33]. Saturation was assessed through research team debriefings following each interview, in which the interviewer and assistant summarized the participants’ answers to the research question. The summaries were continually reviewed during data collection to verify saturation.

The audio recordings were professionally transcribed verbatim. The transcripts were checked for accuracy and deidentified. Interviews were analyzed using a directed content analysis approach [34]: (1) the primary researcher (A.L.M.U.) created a codebook with theoretical codes based on the COM-B framework; (2) the research team (the primary researcher [A.L.M.U.] and one research assistant [H.G.R.]) coded all interviews using the theoretical codes; (3) data labeled with theoretical codes were summarized and informed additional inductive codes that were added to the codebook; (4) transcripts were further coded with inductive codes by the research team; (5) memos were taken through the analysis process to reflect on codes and to document emerging patterns; (6) overall themes and subthemes, based on the codebook and memos, were created. NVivo 12 Plus (QSR International Pty Ltd., Burlington, MA, USA) assisted data organization and analysis. The final codebook is presented in Table 2.

#### 2.4.2. Quantitative Analysis

Demographic survey data was analyzed using descriptive statistics in Microsoft Excel (version 16.49, Microsoft Corporation, 2021, Redmond, WA, USA).

### 2.5. Trustworthiness and Reflexivity

Trustworthiness was established through a team-based approach to coding and data analysis. Two researchers independently coded all interviews and codes were discussed with the research team, enhancing confirmability of findings. In addition, the research team collectively developed the codebook and continually discussed the analysis process and data analysis findings, establishing credibility. Memos were used as reflective notes on the ideas, themes, or connections emerging during the analysis process, further enhancing trustworthiness.

Confirmation bias was address through reflexivity, in which research team members completed journal prompts, followed by group discussions, of how their professional and personal backgrounds, experiences, and prior assumptions are related to the participants’ wider social context and how this might impact interview-participant interactions. Following each interview, the interviewer and research assistant reflected on biases (e.g., if leading questions were asked; if questions that highlighted social or cultural differences between the interviewer and participants were asked; if particular topics were discussed longer than other topics), and whether they influenced the interview. All biases were considered during the analysis and conversations resulting from any biases were eliminated from further analysis.

## 3. Results

Between August 2020 and March 2021 seven video and ten phone interviews were conducted (17 interviews, total). All interviews averaged 38 min in length (range, 24–58 min). Saturation was verified at interview 14, after which three were conducted to ensure no new information would be uncovered.

Participants were, on average, 42.4 ± 12.6 years old. Additional demographic and digital use characteristics are presented in Table 3. All participants (100%) owned a smartphone and 88% used popular apps (Facebook, Twitter, Instagram, Reddit, Snapchat, WhatsApp, Pinterest, or YouTube) “several times a week” or more often.

The following themes were identified regarding apps to deliver ITF education: (1) Most Latina mothers with low-SES are tech-savvy (i.e., high capability and experience using smartphones and apps); (2) Apps are an appealing way to deliver ITF education; (3) There are challenges to using apps that must be carefully considered for ITF education development.

### 3.1. Theme 1: Mothers Are Tech-Savvy

Almost all participants indicated that the majority of Latina mothers are “tech-savvy”: very capable of and experienced with using smartphones, the Internet, and apps. However, many participants indicated that while most were “tech-savvy”, there were some who were not. Participants often associated a mother’s lower English proficiency (and, therefore, inability to read information on websites or follow instructions in English) or lower literacy with low digital literacy. For example, one participant stated:

*“The patients that tend to have a lower health literacy are going to have a lower technology literacy, too. It’s usually an overall knowledge type thing. And it may be a language barrier, but it’s hard to say. I mostly have issues with people that don’t speak English, because they can’t read the app directions.”*

(dietitian)

To overcome these issues, participants emphasized the importance of making an app in English and Spanish and providing mothers with detailed instructions for how to find, download, and use the app. One participant shared their experience of needing to provide detailed instructions when disseminating an app to WIC clients: “We had to record us using the app and giving details of every single click for [mothers] to know… that’s the only thing then, provide the specific and detailed information about the app,” (nutrition educator). Including many visuals was also suggested as an important element of an app; this was thought to make an app more easily understandable to mothers with various Spanish dialects or with low health literacy. For example, this participant emphasized the importance of visuals for educating mothers with various Spanish dialects:

*“Especially with food and feeding, all of the different dialects, depending on where you’re from, it’s very different words. So, a lot of times, even though we have fluent Spanish speakers, if they’re Dominican versus the person that we’re talking to is Guatemalan, sometimes it’s hard. So, I think with actual pictures instead of lots of words, it’s more helpful.”*

(physician)

Nearly all participants believed that smartphone ownership was ubiquitous among Latina mothers, regardless of SES. However, participants held differing opinions on Latina mothers’ Internet access at home, which could impact access to smartphone and app features. Some participants held that nearly everyone had Internet at home. This was especially true of mothers with school-aged children because school was being conducted remotely at the time due to COVID-19. As one participant shared:

*“I was so worried about them. ‘Oh, my God. You have to have Internet, you have to have a phone, you have to do this,’ I had a lot of that, ‘It’s not going to work.’ I was so negative at the beginning. And, let me tell you something. A lot of time I’d be dropping my Zoom meeting, and they are the ones that help me to work through it, so they know everything about apps. I’m so amazed how those moms are now. You know why? Because they had kids in school. They had to learn* *.”*

(nutrition educator)

Additionally, a few physician and dietitian participants noted that they never had issues conducting telehealth visits with Latinx families. Conversely, other participants lamented that many do not have home Internet access or have unreliable access: “You know, a lot of times they don’t have access to reliable forms to get on the Internet either with computers or access to data or Wi-Fi” (physician).

### 3.2. Theme 2: App Appealing for Delivering ITF Education

Most participants deemed an app an effective strategy for creating easy access to valuable ITF education that mothers could use on their own time. As one participant shared, “Nowadays they always use their phones, so I think [an app is] really convenient for mom. Especially when they’re busy, they’re taking care of so many things,” (dietitian). Another participant similarly said:

*“[The] advantage is that you can go as many times as you want during the day. It’s very convenient. ‘Oh, I forgot this. I just sent it to the app or to the website, and it’s there,’ you know? Anytime.”*

(nutrition educator)

Additionally, many viewed an ITF app as a way to capitalize on the frequent and high use of smartphone and apps by Latina mothers.

Participants believed that mothers would value an ITF app if it came from a trusted and reputable source. One participant felt that mothers would prefer having a trusted source of information rather than having to evaluate the legitimacy of information they find online: “There’s so much misinformation on the Internet. Any time there’s a message board where like everyone can see it, I worry about if somebody posts something that’s incorrect, then everybody sees it,” (dietitian). Furthermore, participants often recognized that Latina mothers were eager to learn and apply desirable feeding practices because they believed it would benefit their child, adding to the perceived usefulness of an ITF app. As one participant recounted, “Usually under the age of two, moms are really grateful for [infant and toddler feeding] information. Especially like I said, if they don’t have a foundation of what to do, or how to progress,” (dietitian). Another participant recounted, “The easy part when teaching them is that they’re very open to change. They’re always looking for more. ‘What can I do to be better?’,” (nutrition educator). A few physician and dietitian participants believed that another valuable feature of an app was its potential to connect mothers with a health professional when mothers need counseling rather than being restricted to clinic visits.

Many participants stressed that apps must be culturally relevant, which was thought to make apps more appealing to Latina mothers. As one participant stated, “I think [culturally relevant information] is really important because everybody wants something to apply to them. It just makes you feel seen and understood,” (dietitian). Providing personalized information through an app was also viewed as a motivator to use the app and an effective way to provide information that distinguishes between cultural Latinx subgroups.

### 3.3. Theme 3: Challenges to Using ITF Apps

Despite the many benefits of an app for delivering ITF education to Latina mothers, many participants shared concerns that overall engagement would be mixed. Echoing the opinion of several participants, one participant shared that some mothers would be eager to engage while others simply would not be: “Not everybody is going to be engaged enough to want to download an app,” (physician). A few participants also stressed that initiating use of the app was very important and might be the biggest challenge for engagement: “I think your biggest challenge is going to be marketing and getting them to know about it,” (dietitian). Similarly, another participant shared, “It would be one thing to just say, ‘Well, you can download this app,’ well then, they’ll never do that,” (physician).

Many participants voiced that installing or using a new app would be potentially overwhelming for mothers. Mothers having many apps to choose from added to this feeling: “There’s so many apps and so many things out there that…I don’t know how many people are overwhelmed at this point,” (physician). In reference to existing education, many shared that mothers faced several barriers to enacting recommendations including poverty (food and housing insecurity), conflicting information between healthcare provider and family members, and their child having multiple care takers who did not consistently apply recommendations. Participants suggested that these barriers added to mothers’ feelings of being overwhelmed, which could impact engagement with an app.

Additionally, some participants feared that mothers would experience “information overload,” in which they would be unable to sort through all the information available on an app. For example, one participant said:

*“I also think there’s a point to make where you are providing this app or this website with all of this information, right? Sometimes it could be information overload. And it could be like, ‘I don’t really know where to begin* *.’”*

(dietitian)

As such, many participants warned that including too much information would lead to an app that was not “user-friendly.” For example, one participant shared:

*“You can sometimes run into a quandary when you have to decide how much stuff you’re going to put that your patients have access to without making it so cumbersome or overwhelming that it isn’t user-friendly or [mothers aren’t] able to engage with it.”*

(physician).

In contrast, an app that was “user-friendly” (meaning it was easy to navigate and simple to use), would be beneficial. As one participant stated, “I feel like if [the app is] very user-friendly and there’s not a lot of hoops that you have to jump through, or emails to confirm account. I feel like those things would be beneficial,” (dietitian).

## 4. Discussion

The purpose of this study was to obtain insight on using an app to deliver ITF education to Latina mothers of low SES. Community stakeholders viewed an app as a viable option for providing ITF education to this population. The vast majority of Latina mothers were thought to be “tech-savvy,” indicating they had high capability and extensive experience using smartphones and apps. Indeed, in a recent evaluation of the effectiveness of WIC benefit redemption apps in West Virginia and Kansas, researchers found high voluntary adoption (72.3% of all WIC clients in West Virginia and 80.3% in Kansas) [35], reflecting acceptance of apps among mothers with a low income. Among Latinx ≥18 years old, 98% own smartphones (compared to 93% of the general population) [22], as previously stated, and spend 1.5 h more per week on their smartphone (apps and web browsing) than any other race or ethnic group [36]. This corroborates participants’ belief that smartphone ownership is ubiquitous among Latina mothers of low SES.

Nevertheless, several participants warned that mothers with lower digital literacy, who tended to be Spanish-dominant speakers and had lower health literacy, needed to be taken into account. This highlights that, presently, an ITF app intervention may exclude a segment of mothers in critical need of ITF education. Indeed, less education is associated with accessing the mobile Internet less often among Latinx [37] and with accessing the Internet less often for health information among first-time mothers [38]. If these results apply to Latina mothers, then infants and toddlers of mothers who use mobile technologies less often may be at higher risk of poor health outcomes associated with lower maternal educational attainment (e.g., pediatric obesity and type 2 diabetes mellitus) [39,40]. Given these higher health risks, Latina mothers with lower SES might especially benefit from the additional ITF education and support an app could provide. In terms of English proficiency, Spanish-dominant Latinx individuals often lack full familiarity with using their smartphone and many only use a limited number of apps on their device [41]. However, Spanish-language app interventions have shown success in engaging Latinx participants and improving health-related conditions (e.g., hypertension, diabetes, substance abuse, depression) [42,43,44,45,46,47,48], indicating the feasibility of an app intervention for Spanish-speaking Latinx and resolving the concerns of participants in the present study. In particular, Biediger-Friedman and colleagues found that both English- and Spanish-speaking mothers (82% of whom were Latina) were enthusiastic about a WIC app [27,29]. Strategies to better engage mothers with low digital literacy or who were Spanish-dominant were suggested by participants (e.g., a bilingual app, providing detailed instructions on how to use an app, including more visuals and less text) and should be considered in future programs using apps.

An app was thought to be convenient for Latina mothers and created access to valuable information and support. This is similar to findings of a qualitative study conducted with WIC mothers that found that they desired quick access to reliable and accurate information [29] and were willing to use an app if it was easy to navigate [27]. Participants were enthusiastic about app features that more easily connected mothers to professionals (such as through a message board or texting platform) who could provide ITF education and support. Increased access to virtual information and support is an appealing feature as it is associated with improved health-related factors in similar populations. For example, a mobile-based intervention to prevent obesity among preschoolers that provided information and support from a psychologist or dietitian resulted in improved physical activity levels and diet quality compared to the control group who received pamphlets on healthy eating and physical activity [49]. Salonen et al., found that providing Internet-based support for parenting, breastfeeding, and infant care during pregnancy and early infancy improved parenting satisfaction and self-efficacy [50]. However, whether an ITF education and support app intervention will result in improved infant and toddler obesity rates among Latinx has yet to be investigated. Participants believed mothers would value the information if it came from a reputable source. Whom Latina mothers consider a reputable source within the context of mHealth remains unclear. In qualitative focus groups on intention to use a WIC education app, described above, participants (82% of whom were Latina) indicated trusting information from WIC [27]. However, if other sources would be considered trustworthy was not explored.

Participants often indicated the importance that ITF education for Latina mothers of low SES be culturally relevant. Culturally tailored behavioral health interventions are more effective in improving health outcomes than usual care [51]. Culturally relevant information can enhance an individual’s sense of belonging to a program; as one participant framed it, mothers feel “seen and understood” when information is culturally relevant. In turn, a sense of belonging within the context of health promotion programs has been associated with positive mental and physical health outcomes [52]. The importance of culturally relevant education, especially one that takes into account the heterogeneity of Latinx communities in the United States, may have been especially salient to participants of the present study, because they work in NYC—a city with one of the most diverse Latinx populations among US metro areas [53]. Nationally, Latinx accounted for 52% of population growth between 2010 and 2019 with the fastest population growth occurring among Venezuelans, Dominicans, and Guatemalans [53,54]. As Latinx communities continue to rise in number and diversity, it is increasingly important to take into account the unique needs of mothers with diverse cultural roots from a variety of Latinx countries. An app has the potential to culturally tailor information to the individual, based on information they provide, such as country (or countries) of origin. This type of personalization could, as suggested by participants, increase mothers’ motivation to use the app. Indeed, in a scoping review, researchers noted that the success of mHealth interventions in engaging and improving health outcomes among Latinx demonstrates the importance of culturally tailored interventions and participatory strategies that take into account cultural preferences [55]. Opportunities still remain to further personalize mHealth interventions to meet the specific needs of cultural subgroups within the US Latinx community.

An app presents several challenges to delivering ITF education, as emphasized by participants. Participants felt that Latina mothers may not be interested in an app because they are often overwhelmed with many maternal responsibilities and additional challenges of being from a marginalized community, as highlighted in previous research [56,57]. Many participants were hesitant that mothers would be overwhelmed with receiving too much information, leading to “information overload” and an inability to apply information. This is similar to a qualitative study in which researchers found that mothers felt overwhelmed with the data received from infant feeding tracker apps [23]. Information overload can lead to confusion, misinterpretation of information, and hindered accuracy in decision-making [58]. As such, there was a strong emphasis on apps being “user-friendly.” Likewise, previous research with similar groups of pregnant women and WIC recipients highlighted women’s desire for apps and online sources that are easy to navigate [27,59]. Strategies to accomplish a user-friendly app include user-centered design [60], process evaluation [61], and the Multiphase Optimization Strategy framework [62], allowing researchers to identify the most essential, effective, and popular features.

We considered how the results fit within the COM-B model. For capability, we found that most mothers are highly capable of using apps but may feel too overwhelmed to engage in a novel app. Suggested strategies to overcome mothers feeling overwhelmed was to make apps user-friendly. To increase the capability of mothers with low digital literacy, participants recommended making the app available in English and Spanish, including more visuals and less text, and providing detailed instructions on how to use app. In terms of opportunity, smartphone ownership is ubiquitous but access to Internet (thus limiting features of an app) could be of concern. Strategies to overcome issues of Internet access, thus increasing opportunity, were to make key features available offline. Finally, mothers are believed to be motivated to use an app, as it creates easy access to information that is valued. Strategies to further increase motivation, including making information personalized and culturally relevant, should be considered.

In reflecting on the limitations of the present study, interviews only were conducted with NYC-based health professionals. Therefore, their views may not be generalizable to other cities or states that may face different challenges related to app engagement. Though seven stakeholders who identified as Hispanic/Latinx were interviewed, further research with this group is warranted as their shared culture with Latina mothers could offer more in-depth perspectives on apps not fully captured in this study. Additionally, all but one participant identified as female; therefore, the perspectives of individuals who identify as other genders (e.g., male, gender nonconforming) are needed. COVID-19 could have limited participation of stakeholders because of increasing work demands due to the pandemic or work-related burnout. Corroborating the results of this study with a questionnaire, which may decrease participant burden, could resolve this potential limitation, and will be considered for future research. The different backgrounds and experiential differences of the researchers and participants might have affected the analysis. However, the researchers’ reflection about potential biases throughout the data collection process helped to mitigate this.

The results of this study will be applied to the development of a culturally tailored obesity prevention app intervention for infants and toddlers of Latina mothers of low SES. Prior to the development of the app prototype, it will be essential to gain insight from Latina mothers (both primiparous and multiparous) and family members, such as fathers who play a supportive role in ITF. In particular, it is important to understand perspectives of mothers who are disinterested in mHealth to better understand how to reach this specific population. Key considerations for the development of an app prototype, gleaned from the results of this study, include targeted app promotion; detailed instructions for how to obtain and use the app; including many visuals and being judicious about the amount of written information; options to tailor information to preference of mothers’ Latinx subgroup(s); and making key features available offline.

## Figures and Tables

**Table 1:** Semi-structured interview guide questions with corresponding Capability Opportunity Motivation-Behavior (COM-B) framework construct.

COM-B Construct	Interview Guide Question
Capability	1. How might Latina mothers’ ability to use or access a mobile app ^1^ impact engagement?
Opportunity	2. What are the advantages of using a mobile app ^1^ for infant and toddler feeding education? 3. What are the disadvantages of using a mobile app ^1^ for infant and toddler feeding education?
Motivation	4. How receptive would Latina mothers be to using a mobile app ^1^ to get infant and toddler feeding education?

^1^ App = application.

**Table 2:** Final codebook, with corresponding Capability Opportunity Motivation-Behavior (COM-B) construct, for analysis of interviews with stakeholder participants (*n* = 17).

COM-B Construct	Code	Code Definition
Capability	Language	Ability to use mobile applications related to comprehension of spoken and written language(s).
	Literacy	Ability to use mobile applications related to health literacy level.
	Overwhelmed	Ability to use mobile applications related to feelings of being overwhelmed or psychologically overloaded.
	Digital literacy	Ability to use mobile applications related to ability to use technology (e.g., Internet, smartphones, apps).
Opportunity	Digital access	Access to digital resources (e.g., smartphone ownership, Wi-Fi access, Internet service at home) that facilitate the use of mobile applications.
	User-friendly	Importance or necessity of mobile applications or websites to be easy for mothers to use.
Motivation	Convenience	Mothers’ motivation to use mobile applications because it is a convenient way to access infant and toddler feeding education.
	Engagement	Mothers’ level of engagement with mobile applications.
	Culture	The importance of culture as a way to motivate mothers to use mobile applications.
	Trusted source	The importance of information on mobile applications being recognized by mothers as coming from a trusted source.
	Motivational strategies	Strategies or features that were believed to motivate mothers to start using or continue using mobile applications.

**Table 3:** Sociodemographic and digital use characteristics of stakeholder participants (*n* = 17).

Characteristic	*n* (%)
Gender identity	
Female	16 (94)
Male	1 (6)
Ethnicity	
Hispanic, Latino, or of Spanish origin	7 (41)
Not Hispanic, Latino, or of Spanish origin	10 (59)
Race	
Asian	2 (12)
White	9 (53)
Other	5 (29)
Prefer not to answer	1 (6)
Highest degree obtained	
Some college, no degree	3 (18)
Bachelor’s degree	2 (12)
Master’s degree	8 (46)
Professional degree	3 (18)
Doctorate	1 (6)
Profession	
Nutrition Educator	3 (18)
Physician	5 (29)
Registered Dietitian	9 (53)
Years practicing in primary profession	
3–4	7 (41)
5–9	3 (18)
≥10	7 (41)
Smartphone ownership	17 (100)
Internet use	
Almost constantly/several times a day	17 (100)
About once a day/several times a week	0 (0)
Less often	0 (0)
Prefer not to answer	0 (0)
Use of ≥1 popular apps ^1^	
Almost constantly/several times a day	11 (65)
About once a day/several times a week	4 (24)
Less often	1 (6)
Prefer not to answer	1 (6)
Attitude towards Internet ^2^	
Good thing	11 (65)
Bad thing	0 (0)
Some of both	6 (35)
Prefer not to answer	0 (0)

^1^ Frequency of use was asked about the following popular mobile applications: Facebook, Twitter, Instagram, Reddit, Snapchat, WhatsApp, Pinterest, YouTube. ^2^ Participants responded to the question, “Overall, when you add up all the advantages and disadvantages of the Internet, would you say the Internet has mostly been a good thing or a bad thing for you?”.

## Data Availability

The data presented in this study are available on request from the corresponding author. The data are not publicly available due to the nature of qualitative data, which contains sensitive information and poses a high risk of revealing the identity of study participants.
